# Extracellular Vesicle-Mediated Angiogenesis in Peripheral Artery Disease: Context-Dependent Effects in Ischemic Limbs and Atherosclerotic Plaques

**DOI:** 10.3390/ijms27219725

**Published:** 2026-10-29

**Authors:** Yuya Tamaru, Yusuke Yoshioka, Shinsuke Kikuchi, Takahiro Ochiya

**Affiliations:** 1 Division of Molecular and Cellular Medicine, Institute of Medical Science, Tokyo Medical University, 6-7-1 Nishishinjuku, Shinjuku-ku, Tokyo 160-0023, Japan; tama9222@asahikawa-med.ac.jp (Y.T.); yyoshiok@tokyo-med.ac.jp (Y.Y.); 2 Department of Vascular Surgery, Asahikawa Medical University, 1-1-1 Midorigaokahigashi2-jo, Hokkaido, Asahikawa-shi 078-8510, Japan; kikuchi@asahikawa-med.ac.jp

**Keywords:** peripheral artery disease, atherosclerosis, extracellular vesicles, microRNA, angiogenesis

## Abstract

Peripheral artery disease (PAD) is most commonly caused by atherosclerotic arterial stenosis or occlusion, which reduces downstream tissue perfusion and causes tissue ischemia. In response to ischemia, reparative angiogenesis is activated and may support tissue perfusion recovery and skeletal muscle protection. In contrast, neovascularization within atherosclerotic plaques may promote intraplaque hemorrhage and plaque destabilization. Thus, angiogenesis has dual roles in PAD-related ischemia and atherosclerotic plaques. Extracellular vesicles (EVs) are membrane-bound particles that carry various cargo, including proteins, lipids, nucleic acids, and metabolites. Through the transfer of bioactive cargo and surface-mediated signaling, EVs can modify recipient-cell functions and thereby influence angiogenic signaling. Investigation of EV-mediated communication may therefore help elucidate the mechanisms underlying angiogenic regulation in vascular disease. In addition, disease-promoting EVs may be therapeutically targeted, whereas engineered EVs may be developed as therapeutic agents or delivery platforms. Recent studies have shown that EVs regulate angiogenic responses in preclinical murine hindlimb ischemia models. However, these effects in ischemic limbs and the potential involvement of EVs in atherosclerotic plaque neovascularization have rarely been reviewed in an integrated manner. This narrative review discusses these contrasting angiogenic processes from a context-dependent perspective. Understanding this context dependency may help develop EV-based strategies that promote functional vascular repair in ischemic tissues while suppressing or normalizing pathological neovascularization in atherosclerotic plaques.

## 1. Introduction

Atherosclerosis is a chronic inflammatory disease that occurs in medium- and large-sized arteries, and causes coronary artery disease (CAD), cerebrovascular disease, and peripheral artery disease (PAD) [1,2]. The prevalence of atherosclerosis-related cardiovascular diseases (CVDs) continues to increase. According to the World Health Organization, CVDs caused over 19.8 million deaths in 2022. Among the atherosclerotic diseases, the global number of people aged 40 years or older living with PAD increased from 65.8 million in 1990 to 113.4 million in 2019, while the total disability-adjusted life years (DALYs) attributable to PAD nearly doubled from 0.776 million to 1.536 million during the same period. Thus, PAD is important from both clinical and socioeconomic perspectives [3,4]. PAD is most commonly caused by atherosclerosis and presents a wide clinical spectrum, ranging from asymptomatic disease and intermittent claudication to chronic limb-threatening ischemia (CLTI) [5,6]. The pathophysiology of PAD involves complex intercellular communication among vascular cells, immune cells, and ischemic skeletal muscle cells. Extracellular vesicles (EVs) represent an important mechanism of such intercellular communication. EVs are membrane-bound particles secreted by almost all cell types. EVs are heterogeneous, and terms such as exosomes, ectosomes, microvesicles, and apoptotic bodies have historically been used to describe different EV populations according to their presumed biogenesis and physical characteristics. Exosomes are considered to originate from the endosomal system, whereas ectosomes originate from the plasma membrane and have also historically been referred to as microvesicles or microparticles; however, MISEV2023 discourages the use of biogenesis-based terms unless their subcellular origin has been specifically demonstrated and recommends the generic term “EVs” or operational terms based on measurable characteristics. Although these EV populations share the ability to carry bioactive molecules, they may differ in their physical characteristics, molecular composition, cellular origin, and biological functions, while the heterogeneity of isolated EV populations and the difficulty in establishing their biogenesis often preclude definitive subtype assignment [7]. They mediate intercellular communication through multiple mechanisms, including the transfer of bioactive cargo, such as proteins, lipids, DNA, messenger RNA (mRNA), microRNA (miRNA), long non-coding RNA (lncRNA), circRNAs, and metabolites, as well as signaling mediated by EV-associated surface molecules, thereby modifying cellular phenotypes and functions [8,9]. EV cargo composition changes depending on the cellular environment and disease condition; thus, EVs derived from the same donor cell type may exert distinct biological effects across different contexts, either promoting or suppressing disease progression [9]. In addition, EVs have a lipid bilayer membrane that protects their cargo from degradation by proteases and nucleases [8]. Through the protected transfer of context-dependent cargo, EVs may modulate disease progression and can also be harnessed as vehicles for therapeutic molecules [10,11]. Accordingly, EVs have attracted considerable attention as potential therapeutic targets and platforms in a wide range of diseases, including cancer, inflammatory disorders, neurodegenerative disorders, and atherosclerosis [9,10,12]. In this context, two complementary therapeutic strategies have been investigated: inhibiting the release or uptake of disease-promoting EVs generated in pathological microenvironments, and administering engineered EVs designed to ameliorate disease [10,11].

In atherosclerotic vascular disease, experimental studies have shown that EVs contribute to multiple processes, including endothelial cell (EC) dysfunction, vascular inflammation, immune cell activation, lipid accumulation and foam cell formation, smooth muscle cell phenotypic switching, calcification, and angiogenesis [13,14,15,16,17]. Whereas angiogenesis may support tissue perfusion and repair in PAD-related ischemia, pathological intraplaque neovascularization can promote intraplaque hemorrhage and plaque destabilization [18,19,20].

Despite growing evidence that EVs regulate angiogenesis in these settings, EV-mediated angiogenesis has rarely been systematically reviewed by integrating both PAD-related ischemia and atherosclerotic plaque neovascularization. Such integration is important because EV-mediated angiogenesis cannot be interpreted as uniformly beneficial or detrimental without considering the anatomical and disease setting. This perspective is also clinically relevant because ischemic tissues and atherosclerotic plaques coexist in the same patient with atherosclerotic PAD, raising the possibility that systemic pro-angiogenic EV-based therapies could improve limb perfusion while potentially exacerbating pathological neovascularization within atherosclerotic plaques. Because the available evidence is more extensive for PAD-related ischemia than for plaque neovascularization, this review primarily focuses on EV-mediated angiogenic regulation in PAD-related ischemia, while also discussing atherosclerotic plaque neovascularization as a contrasting pathological context. Understanding this context dependence may help inform the design and safety evaluation of EV-based strategies that promote functional vascular repair in ischemic tissues without exacerbating pathological neovascularization in atherosclerotic plaques. In accordance with MISEV recommendations, this review generally uses the term “EVs” as a generic term; however, terms such as “exosomes,” “microvesicles,” and “microparticles” are retained when referring to the terminology used in the original articles [7,8].

## 2. Literature Search Strategy

A focused narrative search of the literature was performed using PubMed without a predefined date restriction, with the final search performed in March, 2026. We searched combinations of terms related to extracellular vesicles (“extracellular vesicle,” “exosome,” “microvesicle,” and “microparticle”), angiogenesis (“angiogenesis,” “neovascularization,” and “vascularization”), and the relevant diseases or experimental models (“peripheral artery disease,” “limb ischemia,” “hindlimb ischemia,” “atherosclerosis,” “atherosclerotic plaque,” and “vasa vasorum”). We mainly focused on original human, animal, and in vitro studies that were directly relevant to EV-mediated angiogenic regulation. Additional PubMed searches were also carried out to identify background literature on PAD, plaque biology, angiogenic signaling, EV biology, and therapeutic applications. As this was a narrative rather than a systematic review, the studies included were selected based on their relevance to the main topics discussed in this review, rather than through a formal exhaustive screening process. Representative studies were chosen to reflect the major EV sources, classes of EV-associated molecular regulators, experimental models, and mechanistic approaches relevant to the scope of this review.

## 3. Angiogenesis in PAD-Related Ischemia and Atherosclerotic Plaques

### 3.1. Angiogenesis in PAD-Related Ischemia

Arterial stenosis or occlusion reduces tissue perfusion, resulting in hypoxia and nutrient deprivation in tissues [6]. Vascular repair in ischemic limbs involves several distinct processes, including angiogenesis, arteriogenesis, and vasculogenesis. Angiogenesis refers to the formation of new capillaries from pre-existing vessels, whereas arteriogenesis refers to the growth and remodeling of pre-existing collateral arteries. Vasculogenesis refers to de novo blood vessel formation from endothelial progenitor cells. In this review, we primarily focus on angiogenesis as a component of vascular repair in PAD-related ischemia. The resulting hypoxia activates pro-angiogenic signaling pathways, which stimulate EC proliferation, migration, and capillary growth [21]. These processes can improve oxygen and nutrient delivery in ischemic tissues [18,22]. Angiogenic responses in PAD-related ischemia are generally considered adaptive and potentially beneficial because they contribute to tissue perfusion and help preserve tissue viability [21]. However, the functional benefit of angiogenesis in ischemic tissues depends not only on the formation of new vessels but also on their maturation, stabilization, and integration into the existing microcirculation [18,21]. Recruitment of mural cells, including pericytes and vascular smooth muscle cells (VSMCs), contributes to the stabilization of nascent vessels, whereas inadequate mural-cell coverage may leave newly formed vessels immature and poorly functional [18].

In practice, PAD patients often have underlying medical conditions such as diabetes mellitus, chronic kidney disease, and systemic inflammation. These disease-related abnormalities can impair endogenous vascular repair by disrupting endothelial function, reparative-cell activity, and angiogenic signaling. Consequently, angiogenic responses are frequently insufficient [23,24]. Therefore, understanding how angiogenesis is regulated in ischemic limbs is essential for developing strategies to improve tissue repair.

### 3.2. Angiogenesis in Atherosclerotic Plaques

As atherosclerotic plaques progress, inflammatory-cell accumulation and vascular-cell activation increase local metabolic demand, while plaque enlargement and intimal thickening increase the diffusion distance from the arterial lumen, thereby contributing to intraplaque hypoxia [25]. These conditions stimulate angiogenic signaling pathways that promote intraplaque neovascularization from adventitial vasa vasorum through EC proliferation, migration, and sprouting [19,26,27]. This process helps supply oxygen and nutrients to hypoxic plaque lesions. However, within the inflammatory and hypoxic plaque microenvironment, intraplaque neovessels are often immature, structurally fragile, and highly permeable [27,28]. These immature and leaky microvessels promote erythrocyte extravasation, intraplaque hemorrhage, and plaque destabilization [20,26]. In support of this concept, intraplaque hemorrhage has been associated with coronary atheroma progression [29], and immature plaque microvessels have been linked to carotid plaque instability and ischemic symptoms [30]. Therefore, proper vessel maturation and stabilization are critical for determining the biological consequences of plaque neovascularization [20].

### 3.3. The Dual Role of Angiogenesis in PAD-Related Ischemia and Atherosclerotic Plaques

**Figure 1:**
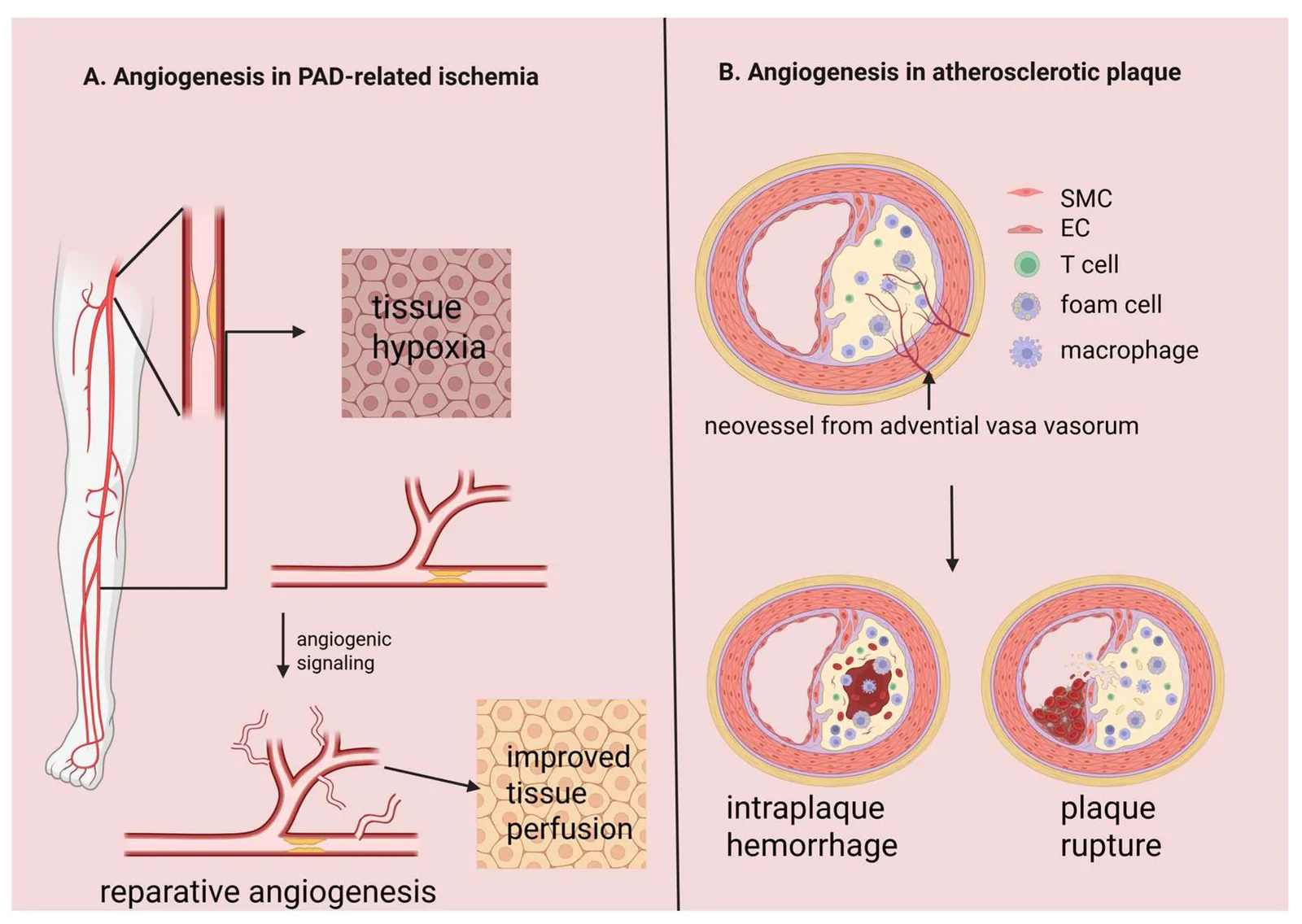
Dual roles of angiogenesis in PAD-related ischemia and atherosclerotic plaque.

The context-dependent contrast between angiogenesis in PAD-related ischemia and atherosclerotic plaque neovascularization is summarized in Figure 1. For the purposes of this review, “vascular quality” refers to the structural and functional characteristics of newly formed vessels, including their maturation and stabilization, mural-cell coverage, permeability, perfusion, and functional integration into the existing vascular network. This dual role indicates that therapeutic strategies should not simply promote or suppress angiogenesis globally but should regulate it according to the anatomical and pathological context. EVs are particularly relevant to this context-dependent regulation because their biological effects are influenced by the microenvironment of donor cells, the identity and functional state of recipient cells, and the surrounding tissue microenvironment [31]. From a therapeutic perspective, EV cargo can be modulated by altering the donor-cell microenvironment, and EV delivery may potentially be targeted to specific tissues or recipient cells [10,11].

In PAD-related ischemia, arterial stenosis or occlusion reduces downstream blood flow, resulting in tissue hypoxia. Hypoxia-induced angiogenic signaling promotes reparative angiogenesis, which can improve tissue perfusion and support tissue repair. In contrast, in atherosclerotic plaques, plaque hypoxia and inflammation promote neovessel formation from the adventitial vasa vasorum. These plaque-associated neovessels are often immature, fragile, and leaky, thereby contributing to intraplaque hemorrhage and plaque destabilization or rupture. Thus, angiogenic responses are context dependent: angiogenesis may be reparative in ischemic limb tissues, whereas intraplaque neovascularization is generally pathological in advanced atherosclerotic plaques. Created in BioRender. 田丸, 祐. (2026) https://BioRender.com/atd0cyi (accessed on 27 October 2026). Abbreviations: EC, endothelial cell; PAD, peripheral artery disease; SMC, smooth muscle cell.

## 4. Extracellular Vesicles as Mediators of Angiogenesis

As described above, EVs mediate intercellular communication through bioactive cargo transfer and surface-mediated signaling and have attracted attention as potential therapeutic targets and platforms. Therefore, in the field of PAD-related ischemia and atherosclerosis, recent studies have focused on EV-mediated angiogenesis [32]. In PAD-related ischemia, evidence has accumulated across experimental settings, ranging from in vitro vascular-cell assays to in vivo murine hindlimb ischemia models. For example, circulating EVs from patients with PAD increased VSMC migration but inhibited EC migration in vitro and contained distinct vascular-active miRNA cargo, indicating that disease-associated EVs can alter vascular-cell behavior [33]. In vivo, multiple studies using murine hindlimb ischemia models have reported that EVs can promote angiogenesis, perfusion recovery, and tissue repair [34,35,36]. However, perfusion recovery in these models reflects not only capillary angiogenesis but also collateral arteriogenesis; therefore, improvements in limb perfusion alone should not be interpreted as direct evidence of angiogenesis. In addition, these models generally induce acute ischemia through surgical arterial occlusion and do not fully reproduce the chronic atherosclerotic disease and complex comorbidities characteristic of human PAD. Thus, although they provide useful mechanistic evidence relevant to PAD-related ischemia, their findings should be extrapolated to human PAD with caution [37]. In contrast, evidence directly linking EVs to angiogenesis within atherosclerotic plaques remains limited and largely indirect, although recent human plaque EV studies suggest potential endothelial communication and angiogenesis-related responses [38]. Recent tissue-EV and plaque-derived EV studies have further shown that human atherosclerotic tissues contain disease-associated EV cargo related to atherosclerosis and plaque vulnerability, although their direct roles in plaque neovascularization remain to be clarified [39,40]. Importantly, the angiogenic effects of EVs are not uniform but vary according to their cellular origin, the pathological state of donor cells, molecular composition, the identity and functional state of recipient cells, and the signaling pathways activated in recipient cells [9,31]. For example, CD34-positive cell-derived EVs carrying miR-126-3p promote reparative angiogenesis in ischemic limbs, whereas skeletal muscle cell-derived EVs enriched in miR-499-5p under diabetic conditions impair endothelial function and delay ischemic limb recovery [34,41]. The following sections first discuss the major classes of EV-associated molecular regulators involved in angiogenic regulation, followed by the downstream signaling pathways implicated in EV-mediated angiogenesis. It should be noted that throughout Section 5 and Section 6, the strength of evidence differs substantially between PAD-related ischemic models and atherosclerotic plaque neovascularization. In brief, evidence for PAD-related ischemia is derived largely from in vivo murine hindlimb ischemia models, whereas evidence relevant to atherosclerotic plaque neovascularization comes mainly from in vitro assays using human plaque-derived EVs and from other indirect experimental models; direct in vivo evidence remains limited. This asymmetry in evidence is a recurring theme and is discussed in detail in Section 8.

Representative studies are summarized in Table 1. Because direct evidence demonstrating that defined EV-associated molecular regulators drive intraplaque neovascularization in vivo remains scarce, this review also summarizes indirect evidence relevant to plaque-associated angiogenesis, including vasa vasorum angiogenesis and endothelial angiogenic responses, in Table 1.

Section A summarizes studies performed mainly in ischemic limb models relevant to PAD-related ischemia. Section B is subdivided according to the proximity of the evidence to atherosclerotic plaque neovascularization. Section B1 includes studies using EVs or microparticles directly isolated from human atherosclerotic plaques and evaluating angiogenic endothelial responses. Section B2 includes experimental studies relevant to plaque neovascularization or vasa vasorum angiogenesis but not directly using plaque-derived EVs.

Abbreviations: ADRCs, adipose-derived regenerative cells; ADSCs, adipose-derived mesenchymal stem cells; AKT, protein kinase B; ASCs, adipose stem cells; cryo-EM, cryogenic electron microscopy; DLS, dynamic light scattering; DM, diabetes mellitus; EM, electron microscopy; eNOS, endothelial nitric oxide synthase; EPCs, endothelial progenitor cells; ERK, extracellular signal-regulated kinase; FACS, fluorescence-activated cell sorting; GLO-1, glyoxalase 1; HCAECs, human coronary artery endothelial cells; HBO, hyperbaric oxygen; hiPSCs, human induced pluripotent stem cells; IGFBP-3, insulin-like growth factor-binding protein 3; iPSCs, induced pluripotent stem cells; iVPCs, induced vascular progenitor cells; JAK, Janus kinase; KLF2, Krüppel-like factor 2; MAPK, mitogen-activated protein kinase; miR, microRNA; MPs, microparticles; MSCs, mesenchymal stem cells; NO, nitric oxide; NRG1, neuregulin 1; NTA, nanoparticle tracking analysis; SEC, size-exclusion chromatography; SEM, scanning electron microscopy; Shh, Sonic hedgehog; STAT6, signal transducer and activator of transcription 6; TEM, transmission electron microscopy; VEGF, vascular endothelial growth factor; VEGFR, vascular endothelial growth factor receptor; WB, Western blotting.

## 5. EV-Associated Molecular Regulators of Angiogenic Signaling

EV-associated molecular regulators, including miRNAs, long noncoding RNAs (lncRNAs), mRNAs, circRNAs, proteins, bioactive lipids, and surface-associated signaling molecules, play a key role in regulating angiogenic signaling [17,34,36,44,54,58,59,60]. This section summarizes these molecular classes in PAD-related ischemia and in experimental contexts relevant to atherosclerotic plaque neovascularization. Because EVs may carry both transcripts encoding angiogenic mediators and the corresponding proteins, some mediators are discussed in more than one subsection. In Section 5, these molecules are organized by molecular class, whereas Section 6 integrates them according to the signaling pathways they regulate.

### 5.1. EV-Associated miRNAs in Angiogenic Regulation

Among EV cargo, miRNAs have attracted particular attention because they can modulate gene-expression programs in recipient cells and reflect changes in the microenvironment of donor cells. This subsection primarily summarizes recent findings on EV-associated miRNAs involved in angiogenesis in PAD-related ischemia. In addition, it discusses the limited evidence related to atherosclerotic plaque neovascularization.

In ischemic models relevant to PAD, several EV-associated miRNAs have been shown to promote reparative angiogenesis and improve perfusion in ischemic limb tissues. For example, human CD34-positive cell-derived exosomal miR-126-3p promotes ischemic limb repair and reparative angiogenesis by suppressing sprouty-related EVH1 domain-containing protein 1 (SPRED1) and modulating angiogenesis-related gene expression [34]. Similarly, miR-126-enriched induced pluripotent stem cell (iPSC)-derived EVs promote angiogenesis, improve perfusion, and enhance functional recovery [42]. Another study showed that EVs derived from adipose-derived mesenchymal stem cells (ADSCs) promoted vascular regeneration and ischemic muscle repair in diabetic hindlimb ischemia. However, the miR-125b-5p–alkaline ceramidase 2 (ACER2) mechanism was functionally examined in skeletal muscle cells rather than in endothelial cells [43]. It should be noted that most of these findings are derived from acute or diabetic hindlimb models rather than human PAD tissues. Moreover, not all EV-associated miRNAs exert pro-angiogenic effects. In diabetic ischemic limbs, skeletal muscle cell-derived EVs enriched in miR-499-5p have been reported to impair endothelial function and delay ischemic limb recovery [41]. This contrasting example underscores the importance of donor-cell state when interpreting the angiogenic significance of miRNA cargo.

In atherosclerotic plaques, evidence for miRNA-mediated regulation of neovascularization is much more limited. Several studies provide indirect evidence relevant to plaque-associated angiogenesis. For example, microvesicles derived from ECs under atherosclerotic conditions are enriched in miR-92a-3p, which is generally considered anti-angiogenic, and these microvesicles modulate EC tube formation in vitro and angiogenesis in Matrigel plug assays through thrombospondin-1 [61]. These findings suggest that EV-associated miRNAs may influence plaque-relevant angiogenic behavior, but they do not demonstrate regulation of intraplaque neovascularization in vivo.

### 5.2. EV-Associated Nucleic Acids Other than miRNAs in Angiogenic Regulation

In addition to miRNAs, EVs contain lncRNAs, mRNAs, and circRNAs, which may regulate angiogenesis through diverse mechanisms; however, their roles in PAD-related ischemia and atherosclerotic plaque neovascularization remain less well defined than those of EV-associated miRNAs. A foundational non-ischemic study showed that endothelial progenitor cell (EPC)-derived microvesicles transferred mRNAs to recipient endothelial cells and activated an angiogenic program [58]. Although this study was not performed in a PAD-related ischemia model, it provides mechanistic support for the concept that EV-associated mRNAs can regulate endothelial angiogenic responses.

In ischemic limb models relevant to PAD, adipose stem cell (ASC)-derived EVs improve vascular growth and skeletal muscle protection [36]. These EVs contain several pro-angiogenic mRNAs, including matrix metalloproteinase-2 (MMP2), fibroblast growth factor 2 (FGF2), VEGFA, and hepatocyte growth factor (HGF). However, functional experiments established a role for neuregulin-1 (NRG1) protein, whereas the individual mRNA species were not directly tested. Another study showed that hyperbaric oxygen (HBO) increased lncRNA metastasis-associated lung adenocarcinoma transcript 1 (MALAT1) levels in EC-derived exosomes. HBO-induced exosomes improved neovascularization in a rat hindlimb ischemia model, whereas the contribution of MALAT1 to angiogenic signaling through the miR-92a/Krüppel-like factor 2 (KLF2) axis was demonstrated mainly in vitro [44]. In addition, EV-associated circRNAs have been implicated in ischemic angiogenesis. For example, exosomes derived from sirtuin 1 (SIRT1)-overexpressing VSMCs contained increased levels of circRNA cZFP609, which was transferred to ECs and reduced hypoxia-induced VEGFA expression by sequestering hypoxia-inducible factor 1α (HIF-1α) in the cytoplasm and thereby preventing its nuclear translocation [54]. In this study, knockdown of cZFP609 in donor VSMCs attenuated these exosome-mediated effects in recipient ECs. Moreover, in vivo cZFP609 knockdown increased capillary and arteriole density and improved perfusion recovery in SIRT1-Tg mice subjected to hindlimb ischemia [54]. In contrast, EPC-derived exosomal hsa_circ_0093884 was shown to promote reparative angiogenesis and improve perfusion in a murine hindlimb ischemia model through the miR-145/SIRT1 axis. Overexpression of hsa_circ_0093884 enhanced these angiogenic effects, whereas its knockdown attenuated them, and the effects were lost in endothelial-specific SIRT1-deficient mice [55]. Thus, available studies support a potential role for EV-associated lncRNAs, mRNAs, and circRNAs in angiogenic regulation in ischemic tissues, although the strength of cargo-specific evidence differs among these RNA classes.

In atherosclerotic plaque neovascularization, direct evidence for EV-associated lncRNAs, mRNAs, and circRNAs is currently lacking.

### 5.3. EV-Associated Proteins in Angiogenic Regulation

EV-associated proteins can also directly regulate angiogenic signaling in recipient cells. In ischemic limb models relevant to PAD, EV-associated proteins have been implicated in reparative angiogenesis. Unlike miRNAs and lncRNAs, which primarily act by modulating gene expression, EV-associated proteins can immediately engage surface receptors or intracellular signaling components in recipient cells. For example, ASC-derived EV-associated NRG1 has been reported to promote vascular growth, tissue repair and skeletal muscle protection in hindlimb ischemia through NRG1/ERBB-associated signaling, including p38 mitogen-activated protein kinase (p38 MAPK) [36]. Sonic hedgehog (Shh)-bearing microparticles derived from activated or apoptotic T lymphocytes promoted post-ischemic neovascularization through activation of the nitric oxide pathway [45].

In contrast, evidence directly linking EV-associated protein cargo to atherosclerotic intraplaque neovascularization remains limited.

### 5.4. EV-Associated Bioactive Lipids and Surface-Associated Signaling Molecules

EV-associated bioactive lipids may also regulate angiogenic signaling. For example, sphingosine-1-phosphate (S1P) associated with platelet-rich plasma-derived exosomes promoted endothelial migration and tube formation through sphingosine-1-phosphate receptor 1 (S1PR1)/protein kinase B (AKT)/fibronectin 1 (FN1) signaling and enhanced angiogenesis in a diabetic wound model mouse [59]. Although this study was not performed in a PAD-related ischemia model, it provides evidence that EV-associated lipid mediators can regulate endothelial angiogenic responses. In addition to intravesicular cargo, EV-associated surface molecules can directly regulate angiogenic signaling. Tissue factor-bearing microparticles released from angiogenic microvascular endothelial cells promoted angiogenesis in vitro and postischemic collateral vessel formation in vivo through β1-integrin-dependent Rac1-ERK1/2-ETS1 signaling and subsequent CCL2 production [60]. In atherosclerotic plaques, CD40 ligand (CD40L)-positive microparticles isolated from human lesions stimulated endothelial proliferation and promoted angiogenesis through CD40-dependent signaling. Their proliferative effects on ECs were attenuated by CD40L or CD40 neutralization and abolished after endothelial CD40 silencing. In mouse Matrigel plug assays, the pro-angiogenic effects were abolished by CD40L neutralization or in CD40-deficient mice [17].

### 5.5. Summary of EV-Associated Molecular Regulators in Angiogenic Regulation

Taken together, among EV-associated molecular regulators, miRNAs have the most extensive evidence base for their roles in angiogenic regulation in both in vitro assays and in vivo murine hindlimb ischemia models. Some EV-associated miRNAs have been shown to promote reparative angiogenesis, whereas others have been reported to impair endothelial function and delay ischemic recovery, suggesting that their effects depend on the state of the donor cells and the disease context. Furthermore, direct in vivo evidence that specific EV-associated molecular regulators influence intraplaque neovascularization remains scarce. Therefore, current evidence is insufficient to establish whether an EV-based intervention can selectively promote functional vascular repair and vessel maturation in ischemic tissues without promoting pathological neovascularization within atherosclerotic plaques. Accordingly, the next section examines the signaling pathways regulated by EV-associated molecular regulators and how disease context may shape the resulting angiogenic consequences.

## 6. Signaling Pathways and Context-Dependent Consequences of EV-Mediated Angiogenesis

**Figure 2:**
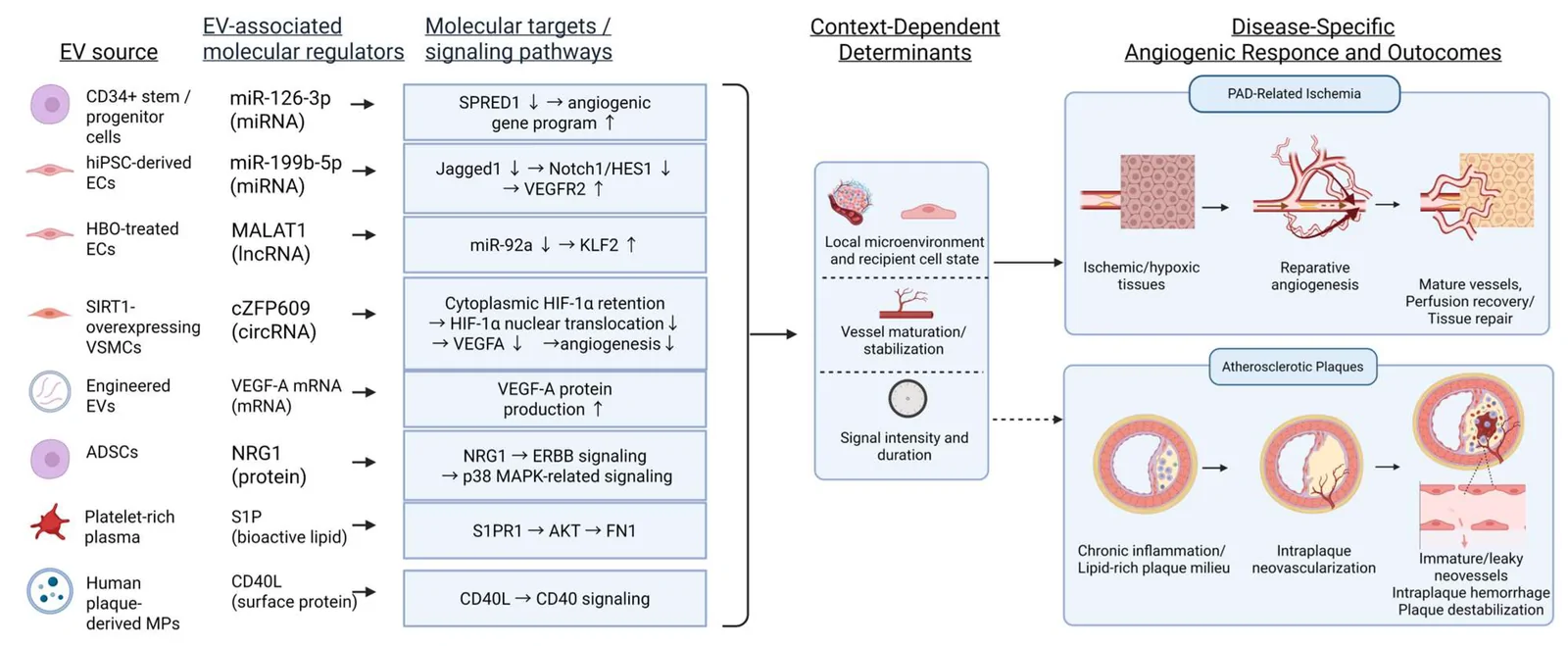
This figure summarizes representative EV sources, EV-associated molecular regulators, and their molecular targets or signaling pathways discussed in this review and illustrates their potential context-dependent vascular consequences. Representative studies show that EV-associated regulators can modulate angiogenic signaling through distinct molecular mechanisms. The downstream vascular outcomes shown in the figure are influenced by context-dependent determinants, including the local microenvironment, recipient-cell state, vessel maturation and stabilization, and signal intensity and duration. The dashed arrow toward atherosclerotic plaque-related outcomes indicates that evidence directly linking defined EV-associated regulators and signaling pathways to intraplaque neovascularization remains limited and predominantly indirect. Therefore, the plaque-related framework is based partly on the broader angiogenesis literature. Representative regulator-target/pathway relationships are shown; additional studies are summarized in Table 1. Created in BioRender. 田丸, 祐. (2026) https://BioRender.com/rlf8q1o (accessed on 27 October 2026). Abbreviations: ADSCs, adipose-derived stem cells; AKT, protein kinase B; CD40L, CD40 ligand; circRNA, circular RNA; ECs, endothelial cells; EVs, extracellular vesicles; FN1, fibronectin 1; HBO, hyperbaric oxygen; HES1, hairy and enhancer of split 1; HIF-1α, hypoxia-inducible factor 1α; hiPSCs, human induced pluripotent stem cells; KLF2, Krüppel-like factor 2; lncRNA, long non-coding RNA; MALAT1, metastasis-associated lung adenocarcinoma transcript 1; MAPK, mitogen-activated protein kinase; miRNA, microRNA; MPs, microparticles; NRG1, neuregulin 1; PAD, peripheral artery disease; S1P, sphingosine-1-phosphate; S1PR1, sphingosine-1-phosphate receptor 1; SIRT1, sirtuin 1; SPRED1, sprouty-related EVH1 domain-containing protein 1; VEGF-A, vascular endothelial growth factor A; VEGFR2, vascular endothelial growth factor receptor 2; VSMCs, vascular smooth muscle cells.

As discussed in Section 5, EV-associated molecular regulators, including miRNAs, lncRNAs, mRNAs, circRNAs, proteins, bioactive lipids, and surface-associated signaling molecules, can regulate angiogenesis. This section first discusses how these regulators modulate angiogenic signaling networks and then examines how the vascular consequences of pathway activation may vary according to disease context. Representative EV-associated regulator–target/pathway relationships summarized in this review are shown in Figure 2.

### 6.1. Angiogenic Signaling Networks Modulated by EVs

Angiogenesis is regulated through the coordinated activity of multiple interconnected signaling pathways rather than by any single pathway acting in isolation. Although substantial crosstalk exists among these pathways, several representative pathways and their predominant functions can be summarized as follows: the vascular endothelial growth factor (VEGF)/vascular endothelial growth factor receptor (VEGFR) axis primarily regulates endothelial activation, migration, and sprouting [62]; phosphoinositide 3-kinase (PI3K)/AKT-related signaling contributes to endothelial survival, migration, and nitric oxide production [63]; MAPK/extracellular signal-regulated kinase (ERK) signaling contributes to endothelial proliferation and migration [64]; and nitric oxide-related signaling contributes to vasodilation, endothelial function, and post-ischemic neovascularization [65]. Several of these pathways are implicated in angiogenic responses in ischemic tissues and contribute to intraplaque neovascularization in atherosclerotic plaques [66,67]. Studies of EV-mediated angiogenesis in murine hindlimb ischemia models relevant to PAD-related ischemia indicate that EVs can modulate multiple components of these interconnected signaling pathways. Representative EV-associated regulator-target relationships include miR-199b-5p-mediated suppression of the Jagged1/Notch1-HES1 axis [46], NRG1/ERBB-associated p38 MAPK signaling [36], and nitric oxide-related mechanisms mediated by Shh-bearing microparticles [45]. Despite these distinct upstream mechanisms, their effects converge functionally on interconnected angiogenic networks involving VEGFR, PI3K/AKT, MAPK/ERK, Notch, and nitric oxide signaling. However, the mechanistic resolution varies among studies. Some studies establish defined EV–associated regulator-target relationships, whereas others report activation of several angiogenic signaling pathways without definitively identifying the responsible EV-associated regulator or direct molecular target. Furthermore, although angiogenic signaling pathways are generally implicated in atherosclerotic plaque neovascularization, direct evidence linking EVs and EV-associated molecular regulators to the activation of specific angiogenic signaling pathways remains limited. Section 6.2 therefore examines how disease context may shape the vascular consequences of EV-regulated angiogenic signaling.

### 6.2. Context-Dependent Consequences of EV-Regulated Angiogenic Signaling

The biological consequences of EV-regulated angiogenic signaling may be highly context dependent. In PAD-related ischemia, activation of angiogenic pathways by EVs or EV-associated molecular regulators may support reparative neovascularization, perfusion recovery, endothelial survival, and skeletal muscle protection [34,35,36]. In contrast, angiogenic signaling pathways have been implicated in intraplaque neovascularization. Plaque neovessels are often immature and leaky, and they may promote inflammatory cell entry, erythrocyte leakage, intraplaque hemorrhage, and plaque destabilization [66,67].

Because EV-specific evidence explaining these divergent vascular outcomes remains limited, the following mechanistic framework is derived partly from the broader angiogenesis literature rather than being directly demonstrated in EV studies of PAD-related ischemia or plaque neovascularization. In ischemic tissues, endothelial sprouting can become reparative when accompanied by mural-cell recruitment and vessel maturation [18]. In brief, functional neovessels improve perfusion and may reduce the initiating hypoxic stimulus. In contrast, intraplaque neovascularization occurs in a hypoxic, macrophage-rich environment in which continued plaque growth and metabolic demand may prevent normalization of tissue oxygenation; moreover, immature and poorly stabilized neovessels facilitate leakage, hemorrhage, and inflammatory-cell entry [25,66,67]. Thus, the outcome depends not only on which pathways are activated but also on whether vascular growth reduces the local hypoxic stimulus and is coordinated with vessel maturation and stabilization. Pathway activation alone may not fully account for these context-dependent outcomes; donor-cell state may shape EV cargo composition, while the phenotype of recipient endothelial cells may influence their responses to EV-mediated signals, thereby affecting vessel quality [31].

Taken together, EV-mediated angiogenesis should be interpreted in relation to disease context and vascular quality rather than simply as pro- or anti-angiogenic.

## 7. Therapeutic Implications of EV-Mediated Angiogenesis

As described above, angiogenesis has opposing implications in ischemic limbs and atherosclerotic plaques. Therefore, therapeutic agents targeting angiogenesis in atherosclerotic vascular disease should be designed in a disease- and tissue-specific manner.

Over the past few decades, therapeutic strategies for ischemic diseases, including PAD, have focused on the administration of pro-angiogenic growth factors such as VEGF, FGF and HGF, as well as gene therapy and cell-based therapies using mesenchymal stem cells (MSCs), EPCs, and bone marrow mononuclear cells [68,69]. Although these approaches have demonstrated promising results in preclinical studies, their clinical efficacy has been inconsistent [70,71,72]. This inconsistency may partly reflect the complex pathophysiology of human PAD, including endothelial dysfunction, metabolic abnormalities, inflammation, skeletal muscle degeneration, and calcification [18,73]. Therefore, activation of a single angiogenic signaling pathway may be insufficient to induce functional vascular regeneration.

In recent years, EVs have attracted increasing attention as cell-free therapeutic agents and delivery platforms for modulating angiogenesis in various diseases [10]. Unlike single-growth-factor approaches, EVs can deliver diverse cargo, including miRNAs, lncRNAs, mRNAs, circRNAs, proteins, and lipids, to recipient cells; thus EVs may modulate multiple angiogenic signaling pathways simultaneously [10,11]. In addition, EVs may reproduce the paracrine effects of cell therapy. Moreover, EVs may reduce some risks associated with direct cell transplantation, such as immune responses, uncontrolled cell proliferation, and tumorigenic risk [11,74]. This capacity to influence diverse biological processes, including endothelial survival and skeletal muscle protection and regeneration, represents a potential advantage in PAD, although the presence of multiple cargo does not itself establish coordinated or consistently beneficial effects.

In ischemic limb models relevant to PAD, as described above, recent studies have shown that EVs derived from MSCs, ADSCs, EPCs, and iPSCs can support reparative angiogenesis and perfusion recovery by enhancing EC proliferation, migration, survival, and tube formation [35,42,43,75]. EV engineering strategies range from the delivery of defined angiogenic cargo to approaches that combine vascular repair with the protection or regeneration of ischemic tissues. For example, VEGF-A mRNA-loaded EVs increased neovascularization and arterial perfusion in a murine femoral artery ligation model and improved left ventricular function after coronary artery ligation [76]. Another study showed that a hydrogel containing VEGF/Transcription Factor EB (TFEB)-engineered EVs promoted angiogenesis, improved tissue perfusion, and protected skeletal muscle in murine hindlimb ischemic models [77]. Moreover, in diabetic hindlimb ischemia, miR-17-5p-enriched EPC-derived EVs have also been reported to improve blood flow, microvessel density, and capillary angiogenesis, while reducing skeletal muscle injury and apoptosis [75]. More recently, engineered EVs derived from human iPSC-derived mesenchymal stem cells were developed to simultaneously promote vascularization and skeletal muscle regeneration in ischemic limbs. These EVs were engineered to enrich multiple pro-angiogenic and pro-myogenic factors, including miR-126, miR-21, miR-296, miR-182, platelet-derived growth factor-BB (PDGF-BB), VEGF, FGF2, and HGF, and were cloaked with platelet membranes modified with an ischemia-homing peptide to enhance delivery to ischemic limbs [78]. In addition, EVs can enhance cell-based therapy. Hypoxic human umbilical vein endothelial cell (HUVEC)-derived EVs have been reported to act as a therapeutic booster to improve the curative potential of ADSCs in a hindlimb ischemia model [79]. Collectively, these studies suggest that some EV-based strategies may influence not only angiogenesis but also nonvascular components of ischemic injury. However, because these effects were demonstrated across heterogeneous studies, current evidence does not establish that EVs consistently provide coordinated control of the multiple pathological components of PAD. Representative therapeutic studies using engineered, cargo-loaded, targeted, or donor-cell-conditioned EVs for ischemic limb repair are summarized in Table 2.

In contrast, angiogenesis in atherosclerotic plaques is generally considered pathological because intraplaque neovessels are often immature, fragile, and leaky. Therefore, the therapeutic goal in atherosclerotic plaques should not simply be to suppress angiogenesis but to selectively modulate or normalize pathological intraplaque neovascularization [80]. Previous studies have attempted to suppress angiogenesis using anti-angiogenic approaches, including anti-VEGF therapies. Anti-angiogenic interventions have produced context-dependent effects. Inhibition of plaque neovascularization reduced macrophage accumulation and atherosclerotic lesion progression in some models [81], whereas systemic VEGF inhibition accelerated atherosclerosis and disrupted endothelial homeostasis in another model [82]. Moreover, VEGFR2 blockade has been reported to reduce intraplaque hemorrhage by promoting plaque neovessel maturation [80]. In addition, in patients with atherosclerotic PAD, systemic inhibition of angiogenesis may impair adaptive neovascularization and tissue repair in ischemic regions [18]. Therefore, therapeutic strategies should promote reparative angiogenesis in ischemic tissues while selectively modulating or normalizing pathological intraplaque neovascularization. However, direct evidence that EV-based interventions can achieve this tissue-selective regulation remains scarce. Achieving these context-selective outcomes may require tissue- or lesion-targeted delivery together with cargo designs that promote vascular maturation and tissue repair rather than endothelial sprouting alone. At present, however, this remains a therapeutic design objective rather than an established property of EV-based interventions.

**Table 2:** Engineered and modified EV-based therapeutic strategies for ischemic limb repair. Abbreviations: BDNF, brain-derived neurotrophic factor; FGF21, fibroblast growth factor 21; PTEN, phosphatase and tensin homolog; ROS, reactive oxygen species; TGF-β1, transforming growth factor beta 1.

Engineering/Modification	Therapeutic Cargo or Feature	Disease Model	Main Therapeutic Outcome	Proposed Mechanism	Reference
Engineered EVs	VEGF-A mRNA	Murine femoral artery ligation model; coronary artery ligation model	Improved arterial perfusion in ischemic limbs; improved cardiac function after myocardial ischemia	Local VEGF-A expression promotes angiogenesis	[76]
Engineered EVs	VEGF and TFEB	Murine critical limb ischemia/hindlimb ischemia model	Enhanced angiogenesis; improved tissue perfusion; protected skeletal muscle	VEGF-mediated angiogenesis; TFEB-related cytoprotective and autophagy-related pathway	[77]
GLO-1-overexpressing ADSC-derived EVs	Not defined	Type 2 diabetic mice with limb ischemia	Protected endothelial cells; enhanced angiogenesis; improved ischemic limb recovery	anti-glycation/oxidative stress-related mechanism; detailed EV cargo pathway not fully defined	[47]
Engineered EV derived from EPCs	miR-17-5p	Diabetic hindlimb ischemia model	Improved blood flow; microvessel density; capillary angiogenesis; protected skeletal muscle; reduced apoptosis	SPRED1; PI3K/AKT-related signaling	[75]
Hypoxic HUVEC-derived EVs	miR-486-5p	Limb ischemia model; ADSC therapeutic booster strategy	Enhanced ADSC resistance to ROS; improved ADSC pro-angiogenic ability	miR-486-5p /PTEN axis	[79]
Engineered iMSC-derived EV	upregulated miR-126; miR-21; miR-296; miR-182; PDGF-BB; VEGF; FGF2; HGF	hindlimb ischemia	Restored blood perfusion; Promoted skeletal muscle regeneration	Combined pro-angiogenic/ pro-myogenic cargo enrichment	[78]
PDGF-BB-stimulated ASC-derived EVs	Enriched in TGF-β1; VEGFs; BDNF; FGF21; lncRNA MALAT1	hindlimb ischemia model	Improved perfusion recovery; Reduced ischemic muscle injury; Decreased inflammatory cell infiltration	Not defined	[83]

## 8. Limitations and Future Perspectives

Several limitations remain in the field of EV-mediated angiogenesis. First, general challenges in EV research extend beyond the field of angiogenesis. The MISEV2018 and MISEV2023 guidelines provide various recommendations on EV nomenclature, separation, and characterization methods, and quantitative assessment [7,8]. However, adherence to these recommendations varies across studies. Therefore, caution is required when interpreting these findings because several studies use the term “exosome” despite the lack of definitive evidence. In addition, isolation methods vary among studies and EV characterization is insufficient in some reports. These methodological differences may lead to inconsistent interpretation across studies [7,8,84]. Furthermore, EV populations are heterogeneous, and isolated EV preparations may contain vesicles with different biogenesis pathways and cargo compositions as well as co-isolated non-vesicular material [85,86]. Previous reassessments of exosome composition have also suggested that some molecules previously regarded as exosomal cargo may actually be associated with non-vesicular fractions rather than EVs [85,86]. Therefore, rigorous EV isolation and characterization, together with appropriate controls for co-isolated material, are necessary to determine whether the observed angiogenic effects are attributable to EVs.

Second, the available evidence differs substantially in both strength and nature between PAD-related ischemia and intraplaque neovascularization. Specifically, evidence for EV-mediated angiogenesis in PAD-related ischemia is derived predominantly from acute murine hindlimb ischemia models, whereas direct in vivo evidence linking EVs to intraplaque neovascularization remains limited. Conventional murine hindlimb ischemia models induce acute arterial occlusion surgically in otherwise relatively healthy animals with largely preserved endogenous repair capacity [37]. In contrast, human PAD develops chronically through atherosclerotic stenosis or occlusion and commonly occurs in the presence of comorbidities and pathological conditions such as diabetes, aging, endothelial dysfunction, chronic inflammation, and skeletal muscle degeneration [6,22,23,73]. Therefore, the therapeutic effects observed in hindlimb ischemia models may partly reflect enhancement of a relatively intact endogenous repair response and may not fully reproduce the complex ischemic, inflammatory, metabolic, and degenerative microenvironment of human PAD. Consequently, direct extrapolation of these findings to chronic human PAD may overestimate therapeutic efficacy. Moreover, perfusion recovery in these models reflects both capillary angiogenesis and collateral arteriogenesis and should not be attributed to angiogenesis alone [21,37]. In the context of intraplaque neovascularization, a recent human carotid plaque study combined EV molecular profiling with evidence of endothelial communication and in vitro endothelial sprouting [38]. A separate tissue-EV multiomic study identified disease-associated cargo in human atherosclerotic carotid tissue but did not directly examine angiogenesis [40]. However, these studies are primarily descriptive or based on ex vivo and in vitro observations and do not establish whether defined EV sources or EV-associated molecular regulators causally regulate intraplaque neovascularization in vivo.

Third, because EV-mediated angiogenesis may have opposing consequences in PAD-related ischemic tissues and atherosclerotic plaques, future therapeutic development should be guided by two complementary criteria: tissue selectivity and vascular quality.

EV-associated molecular regulators that promote reparative angiogenesis in ischemic limbs may be beneficial for perfusion recovery and tissue repair, whereas similar pro-angiogenic signals could theoretically promote immature neovascularization, vascular leakage, intraplaque hemorrhage, and plaque destabilization within atherosclerotic plaques [20,26,80]. Future studies should develop disease-relevant experimental models and EV-based therapeutic strategies designed to achieve tissue selectivity and appropriate vascular quality. For model development, PAD-related ischemia models should more closely reproduce the chronic inflammatory, vascular, microcirculatory, and skeletal-muscle abnormalities of human PAD. In parallel, in vivo models capable of directly assessing the role of EVs in intraplaque neovascularization are needed. Where feasible, models that permit concurrent assessment of functional vascular repair in ischemic tissues and intraplaque neovascularization would allow the local and systemic effects of the same EV intervention to be evaluated within a common experimental framework. For therapeutic development, tissue selectivity may be enhanced through targeted delivery strategies. From this perspective, EVs could potentially be surface-engineered to preferentially target ischemic tissues [78], although whether such designs can minimize uptake by plaque-associated cells remains unknown. Alternatively, context-selective therapeutic effects might be achieved by engineering or selecting EV cargo that promotes reparative angiogenesis in ischemic tissues while minimizing pro-angiogenic activity within atherosclerotic plaques. Ultimately, combining such cargo design with tissue-selective delivery may further improve therapeutic efficacy while reducing pathological angiogenesis in atherosclerotic plaques. Therapeutic evaluation should consider not only angiogenic activity but also vascular quality, including vessel maturation, stability, permeability, and functional integration. Future studies should adopt outcome measures that more closely reflect human PAD, including perfused-vessel density, mural-cell coverage, vascular stability and permeability, skeletal muscle structure and function, tissue repair, and the durability of therapeutic effects. The same studies should assess plaque neovessel density and permeability, intraplaque hemorrhage, inflammation, and plaque stability.

## 9. Conclusions

The central message of this review is that angiogenesis should be understood as a context-dependent process; therefore, EV-mediated angiogenesis should not be interpreted simply as beneficial or detrimental. The findings reviewed here suggest that the efficacy of angiogenic therapy in atherosclerotic PAD cannot be defined solely by local perfusion recovery in the ischemic limb. Rather, therapeutic success should be evaluated by balancing reparative effects in ischemic tissues against the potential to promote intraplaque neovascularization and plaque destabilization. From this perspective, the objective of EV-based therapy is not to maximize angiogenic potency but to achieve effective ischemic tissue repair without aggravating pathological intraplaque neovascularization or plaque destabilization. Because their cargo and delivery properties can be engineered, EVs represent a candidate platform for pursuing this balance, although current experimental systems are not yet sufficient to determine whether it can be achieved. Future studies should therefore move beyond isolated hindlimb efficacy testing and evaluate both the beneficial and adverse vascular consequences of the same EV intervention in disease-relevant models that ideally permit concurrent assessment of ischemic tissue repair and intraplaque neovascularization. Ultimately, translation of these findings to human PAD will require validation in well-characterized human studies.

## Figures and Tables

**Table 1:** Representative studies of EV-mediated angiogenic regulation in PAD-related ischemia and atherosclerotic plaque-related contexts.

EV Source	EV Isolation; Characterization	EV-Associated Regulator/ Pathway Target	Model	Vascular Outcome	EV Specific Evidence	Reference
A. EV-mediated angiogenic regulation in PAD-related ischemia / ischemic limb models						
CD34-positive stem/ progenitor cells	ultracentrifugation, sucrose gradients; DLS, flow cytometry, EM, immunoblotting	miR-126-3p/SPRED1 suppression →angiogenesis-related gene activation	hindlimb ischemia	Improved ischemic limb perfusion; increased capillary density; improved motor function	in vivo; cargo specific loss-of-function; target/pathway validation	[34]
iPSCs	ultracentrifugation; NTA, TEM, high-resolution flow cytometry	miR-126/Not defined	acute limb ischemia	Improved limb perfusion and functional recovery; enhanced endothelial angiogenic activity in vitro	in vivo; engineered cargo enrichment	[42]
ADSCs	ultracentrifugation; NTA, TEM, WB	miR-125b-5p/ ACER2 suppression	Diabetic hindlimb ischemia	Improved limb perfusion; increased capillary and arteriole density; enhanced muscle repair	in vivo; cargo-target validation in vitro	[43]
HBO-treated HCAECs	polymer-based precipitation; WB	lncRNA MALAT1/miR-92a suppression→KLF2 upregulation	hindlimb ischemia	Improved ischemic limb blood flow; increased endothelial proliferation and tube formation in vitro	in vivo; cargo/ pathway perturbation	[44]
Activated/ apoptotic human T lymphocytes	differential centrifugation; not reported	Shh protein/NO pathway →eNOS/AKT activation and VEGF-related gene regulation	hindlimb ischemia	Improved limb perfusion; increased CD31+ vascular density	in vivo; pathway inhibition	[45]
MSCs	ultracentrifugation, iodixanol density-gradient ultracentrifugation; NTA, TEM, WB	VEGF protein, miR-210-3p/ VEGFR1/VEGFR2 upregulation→Src/AKT/ERK activation	hindlimb ischemia	Improved blood reperfusion; increased new blood vessel formation	in vivo; cargo identified; pathway shown in vitro	[35]
hiPSCs-derived ECs	ultracentrifugation; NTA, TEM, WB	miR-199b-5p/Jagged1/ Notch1/HES1 suppression →VEGFR2 upregulation	hindlimb ischemia	Improved limb perfusion; increased CD31+ capillary density	in vivo; cargo-specific loss-of-function; pathway validation in vitro	[46]
GLO-1-overexpressing ADSCs	differential centrifugation; NTA, TEM, WB	Not defined/ Not defined	hindlimb ischemia	Improved limb perfusion; increased microvessel density; preserved muscle integrity	in vivo; no cargo specific validation	[47]
ADSCs	ultracentrifugation; flow cytometry, TEM	Not defined/ Not defined	hindlimb ischemia	Improved blood-flow recovery; increased new vessel formation; reduced limb necrosis grade	in vivo; no cargo/pathway validation	[48]
ASCs	ultracentrifugation; NTA, FACS, TEM, WB	NRG1 protein/NRG1 protein-ERBB signaling →p38 MAPK-related signaling	hindlimb ischemia	Increased capillary density; reduced muscle damage/ inflammation; no significant improvement in large-vessel reperfusion	in vivo; cargo-specific inhibition	[36]
ADRCs	ultrafiltration, ultracentrifugation; not reported	Not defined/ Not defined	hindlimb ischemia	Exosome inhibition impaired ADRC-mediated limb perfusion recovery	in vivo; EV biogenesis inhibition; indirect EV involvement	[49]
EPCs	ultracentrifugation; TEM, SEM, FACS	miR-126, miR-296/ Not defined	hindlimb ischemia	Enhanced limb perfusion; Increased capillary density in ischemic gastrocnemius muscle	In vivo; RNA-dependent effects; miRNAs not individually validated	[50]
hiPSCs derived MSCs	ultrafiltration, sucrose cushion- ultracentrifugation; TEM, WB	Not defined/ angiogenesis- related molecules upregulation	hindlimb ischemia	Improved blood perfusion; increased microvessel density; reduced limb loss	in vivo; pathway-associated changes shown in vitro	[51]
ADSCs	ultracentrifugation; NTA, TEM, WB	Not defined/ JAK/STAT6 mediated macrophage M2 polarization	Type 2 DM hindlimb ischemia	Improved blood perfusion; increased microvessel density	in vivo; pathway association shown in vitro	[52]
iVPCs	ultrafiltration, SEC; NTA, TEM, WB	miR-143-3p, miR-291b, miR-20b-5p, IGFBP-3, pentraxin-3 /Not defined	hindlimb ischemia	Improved blood perfusion recovery; increased CD31+ capillary area	in vivo; cargo profiling; not individual cargo validation	[53]
SIRT1- overexpressing VSMCs	polymer-based precipitation; not reported	circRNA cZFP609/HIF-1α nuclear translocation suppression→VEGFA downregulation	hindlimb ischemia	Impaired limb perfusion recovery; cZFP609 knockdown increased capillary and arteriole density and improved perfusion	in vivo; cargo-specific knockdown; pathway validation in vitro	[54]
EPCs	ultrafiltration, polymer-based precipitation; NTA, TEM, WB	circRNA hsa_circ_0093884/miR-145 suppression→SIRT1 upregulation	hindlimb ischemia	Improved limb perfusion; increased capillary density and endothelial angiogenic activity	in vivo; cargo-specific gain/loss-of-function; pathway validation	[55]
B. EV-mediated angiogenic regulation in atherosclerotic plaque-related contexts						
B1. Studies using EVs or microparticles directly isolated from human atherosclerotic plaques and evaluating angiogenic endothelial responses						
Human atherosclerotic plaque	Centrifugation; flow cytometry	CD40 ligand/ CD40-CD40 ligand signaling	EC assay, Matrigel angiogenesis assay	Stimulated endothelial proliferation; promoted neovessel formation in vivo in Matrigel	human plaque-derived MPs; in vivo angiogenesis assay; pathway inhibition	[17]
Human carotid plaque	SEC; NTA, cryo-EM, WB	Multiomics EV cargo profiles/Not defined	Endothelial spheroid sprouting assay	Induced Endothelial sprouting	human plaque-derived EVs; in vitro sprouting assay; multiomic cargo profiling	[38]
B2. Relevant to plaque neovascularization or vasa vasorum angiogenesis but not directly using plaque-derived EVs						
Insulin- resistant adipocytes	ultracentrifugation; NTA, TEM, WB	Shh protein/ Shh signaling	Diabetic ApoE−/− mice	Increased vasa vasorum angiogenesis; plaque burden and vulnerability	in vivo; Shh cargo manipulation; pathway inhibition	[56]
Oxidative stress-activated ECs	ultracentrifugation; NTA, TEM, WB	Not defined/ decreased miR-92a-3p in recipient cells→tissue factor upregulation	In vitro Matrigel assay	Induced endothelial proliferation; migration; tube formation;	in vitro; recipient-cell miR-92a-3p gain/loss-of-function; direct target validation	[57]

## Data Availability

No new data were created or analyzed in this study. Data sharing is not applicable to this article.
